# Usability Evaluation of a Mobile Monitoring System to Assess Symptoms After a Traumatic Injury: A Mixed-Methods Study

**DOI:** 10.2196/mental.5023

**Published:** 2016-01-11

**Authors:** Matthew Price, Tyler Sawyer, Madison Harris, Christian Skalka

**Affiliations:** ^1^ Center for Research on Emotion, Stress, and Technology Department of Psychological Science University of Vermont Burlington, VT United States; ^2^ Department of Computer Science College of Engineering and Mathematical Sciences University of Vermont Burlington, VT United States

**Keywords:** mobile phone, trauma, posttraumatic stress disorder, usability

## Abstract

**Background:**

Victims of trauma are at high risk for mental health conditions such as posttraumatic stress disorder and depression. Regular assessment of mental health symptoms in the post-trauma period is necessary to identify those at greatest risk and provide treatment. The multiple demands of the acute post-trauma period present numerous barriers to such assessments. Mobile apps are a method by which to overcome these barriers in order to regularly assess symptoms, identify those at risk, and connect patients to needed services.

**Objective:**

The current study conducted a usability evaluation of a system to monitor mental health symptoms after a trauma. The system was developed to promote ease of use and facilitate quick transmission of data.

**Methods:**

A sample of 21 adults with a history of trauma completed a standardized usability test in a laboratory setting followed by a qualitative interview.

**Results:**

Usability testing indicated that the app was easy to use and that patients were able to answer several questions in less than 1 minute (mean [SD] 29.37 [7.53]; range 15-57). Qualitative analyses suggested that feedback should be included in such an app and recommendations for the type of feedback were offered.

**Conclusions:**

The results of the current study indicate that a mobile app to monitor post-trauma mental health symptoms would be well received by victims. Personalized feedback to the user was identified as critical to promote the usability of the software.

## Introduction

Approximately one in three victims of a traumatic injury will develop a chronic mental health disorder including posttraumatic stress disorder (PTSD) within 1 year after the trauma [1,2]. Victims of trauma often experience a range of symptoms in the acute post-trauma period [3] that may serve as early indicators of long-term chronic outcomes. Given the trauma is a known event, early intervention delivered shortly after exposure can prevent these outcomes [4]. Indeed, several studies have shown that brief interventions that begin within hours to days after the trauma can mitigate early distress and prevent long-term psychopathology [5,6]. Such early interventions address a major public health concern [7] as PTSD is associated with persistent functional impairment, even in those who have resolved symptoms [8]. Furthermore, relatively few victims of trauma independently seek out mental health treatment in the acute aftermath of a trauma [1,9], which highlights the need for protocols that begin within an acute care setting when patients can be engaged in care while receiving treatment for their specific event.

There are several barriers, however, in implementation of such early interventions [4]. First, it is unclear who is at risk for PTSD immediately after a trauma, such that repeated assessments are necessary [10,11]. Conducting such assessments with interviewers is costly and burdensome [12]. Second, the clinical presentation of patients varies greatly in the acute post-trauma period and in those with chronic presentations of the disorder [13]. Effective early intervention requires targeting a patient’s specific clinical needs [3]. Third, rates of refusal for treatment that begins within hours of the injury and attrition rates for those that engage in such treatment are high [6,14]. Finally, the considerable clinical demands of acute care centers often limit the type of treatment available. Technological solutions, such as mobile apps, have the potential to overcome these barriers, reduce provider burden, and facilitate critical early post-trauma intervention [15]. Indeed, similar monitoring strategies have been accepted for monitoring depression in outpatient clinical settings [16,17], but none has been evaluated for addressing early symptoms that may lead to PTSD in acute care settings.

Mobile apps can advance acute post-trauma care and mental health treatment more broadly [18]. Mobile devices are near ubiquitous among adults in the United States [19]. Approximately half of American adults have downloaded apps to their mobile phones. Health apps can provide education and intervention, facilitate communication between patients and providers, and provide disorder-specific feedback. Communication with patients and providers can occur asynchronously to accommodate patient and provider schedules. Mobile apps are easily disseminated, low cost, and easily integrated with electronic medical records [20]. Finally, mobile apps can be tailored to assess the wide range of possible post-trauma mental health symptoms and those of related conditions. This flexibility is important given that post-trauma symptoms develop at different rates after a trauma [21,22].

In order for mobile app post-trauma care to have the proposed impact on health care, it is necessary to design systems that address the needs of this patient population. Those recently exposed to a trauma have multiple competing concerns in the acute aftermath of an event that place significant demands on their time [3]. Apps created by the Veterans Health Administration and the Department of Defense for chronic PTSD were well received by patients [23,24] and providers [25]. However, these apps may be inappropriate for use in the acute post-trauma period given that symptoms may not have fully developed. Indeed, PTSD symptoms fluctuate in the post-trauma period [21]. Relatedly, the concerns of the patient are likely to vary during the acute period. The best method to ensure that an app addresses the concerns is with a usability evaluation [26]. The belief is that an assessment method should place minimal burden on the patient so as not to interfere with recovery. The mobile app should allow question content to change during the assessment period to capture the course of symptoms. Including a method to capture the dynamic concerns of the patient so intervention and assessment can be tailored accordingly is necessary. To determine if these features are useful in post-trauma care, a mixed-method usability evaluation is needed. The current study conducted a usability evaluation of a system that includes a mobile app to monitor post-trauma symptoms. The primary aim of the current study was to highlight key usability and design components of this platform that will inform development of systems designed to track patient progress.

## Methods

### Participants

A total of 21 college-aged adults with a history of a trauma exposure that resulted in a hospital visit participated in the study. Participants were 19 years old (mean [SD] 18.8 [0.87]), and the majority were female (16/21, 76%) and White (15/21, 71%). All participants owned a smartphone, primarily iPhones (15/21, 71%). Participants all texted, took pictures, listened to music, downloaded apps, recorded videos, and accessed the Internet on their phones. A majority used their devices to obtain information about physical health (17/21, 81%) and mental health (14/21, 67%). All participants provided verbal consent as the Institutional Review Board did not require written consent. Consent was recorded using a required documentation form.

### Development of the Mobile App

A development team with expertise in mobile app development, database creation, acute trauma care, and post-trauma mental health care created a prototype app. Design was guided by the Technology Acceptance Model (TAM) [27], which posits that adoption and continued use of software is a function of perceived usefulness and perceived ease of use. Perceived usefulness is the extent that a technology will increase the likelihood of a given outcome. Applied to the current problem, monitoring may improve the likelihood that an individual receives mental health care after a trauma. Perceived ease of use is the extent that minimal effort is needed to use the technology.

**Figure 1:**
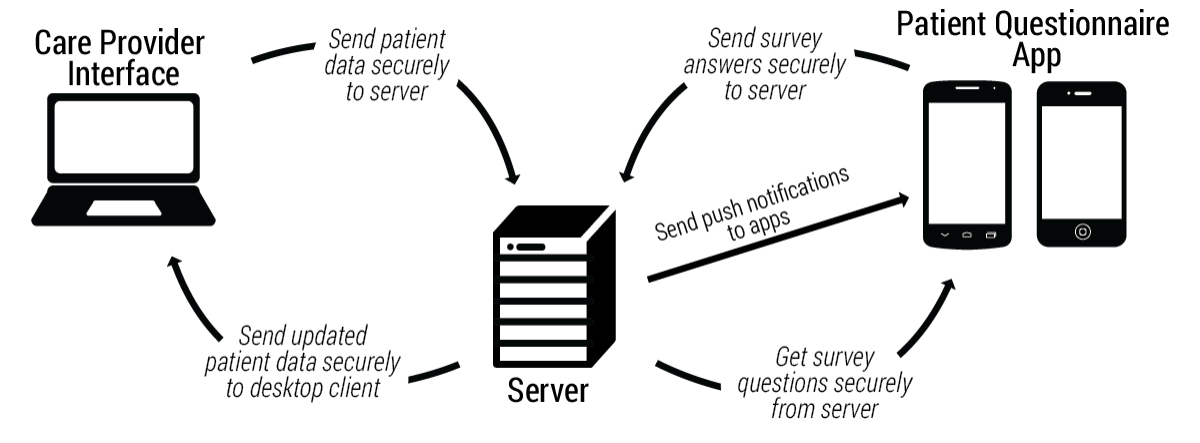
Schematic diagram of the components of the system used to monitor symptoms after a trauma.

A distributed system comprising several major software components was created (Figure 1). The patient-facing component is a mobile app that administers self-report assessments. The system also includes a database and a Web interface for care providers. The Web interface allows providers to manipulate patient data (add new patients, view existing patients, obtain reports of responses provided by patients) and manipulate questions (create, edit, and delete). Creation and modification of questions can be done quickly and efficiently with a series of menus and text fields through the Web portal. Notifications can be assigned to alert the patient to complete an assessment at a specific time or randomly within a pre-specified interval [28]. Notifications are automatically pushed to the mobile app. The following areas were prioritized during the development process.

#### Speed of Completing Assessments

**Figure 2:**
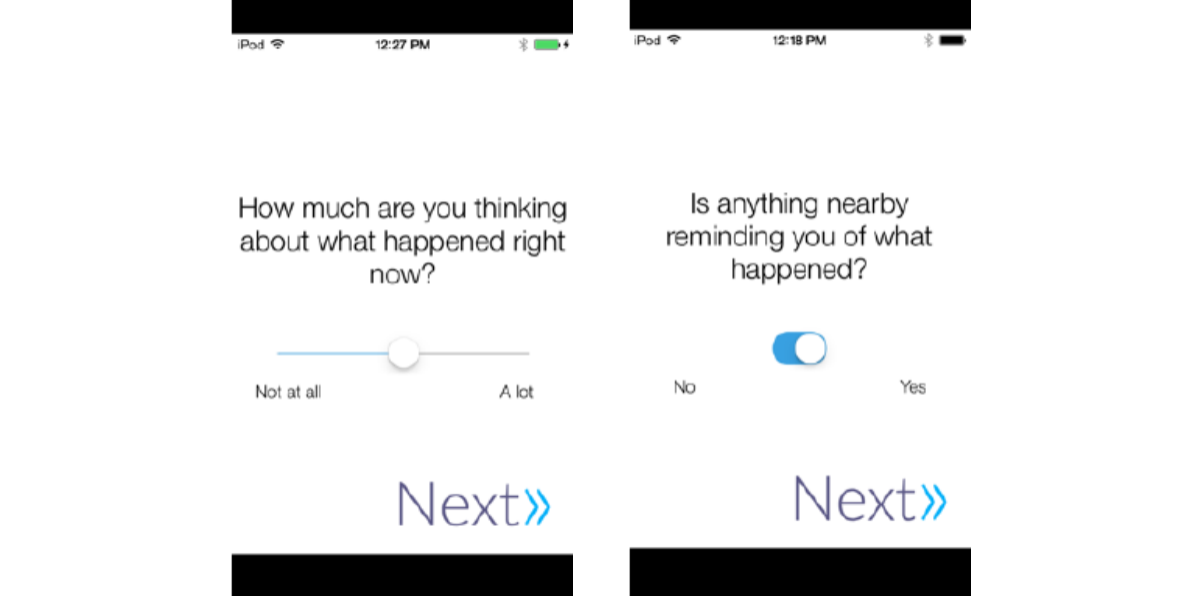
Screenshot of slider and toggle type questions.

Several steps were taken to ensure question sets could be completed quickly. First, two types of question responses were implemented: Sliders and Toggles (Figure 2). Sliders were visual analogue scales, a commonly used method to assess symptom severity in health research [29]. A slider allows participants to make choices more rapidly than other commonly used methods such as a Likert scale. Mobile device screen size limits the amount of text that can be presented, which imposes a challenge presenting a question and corresponding text for 5-9 discrete options on a single screen. Toggle questions were used to ask question with a dichotomous response (eg, Yes/No).

Second, app speed was prioritized. Initial prototypes included multiple icons on the home page that ultimately interfered with speed of use. A home page with a single icon “Begin Questions” in the center was used instead. Transitions between screens were removed. Responses were stored locally on the device and transmitted when the assessment was completed, to eliminate network latency that is common in Web forms [30]. Responses were sent automatically rather than prompting the user to upload their responses.

#### Ease of Use

The types of questions allowed were selected to improve the ease of use. Consideration was given to presenting multiple items on a single screen in which users would scroll through all items or presenting multiple screens. The use of a native app, as opposed to a Web survey, reduced load times to overcome the limitation of using multiple screens to complete a survey [30].

#### Flexibility in Assessment Content

Considerable flexibility was needed to assess a range of symptoms. For example, there are 20 possible symptoms that make up the diagnosis for PTSD. Surveys were allowed to be of unlimited length, have editable content, and have additional items added or removed via the Web interface. Furthermore, participants could be assigned different surveys based on the time of day.

#### Usability Measures

Usability was assessed with the Perceived Useful and Ease of Use Survey (PUEU) [27] and a qualitative interview. The PUEU is a 12-item self-report survey with subscales assessing the perceived usefulness of a given technology (eg, “The app would enable me to communicate with my doctor more quickly”) and the perceived ease of use of a given technology (eg, “It would be easy for me to become skillful at using the system”). Each item is rated on a 7-point Likert scale with higher scores indicating a more favorable rating. A qualitative interview was conducted to assess impressions of the app and guide subsequent development. Participants were asked for their thoughts on the app, components they liked most, components they liked least, and to suggest features to improve the app. Responses were audio recorded and transcribed for review.

#### Mobile Devices

The mobile app was evaluated on an iOS (iPod Touch 5^th^ Gen) and an Android device (Motorola Moto G). The interface was nearly identical across both platforms. Half of the sample used each device. Use of the mobile app was monitored using a universal serial bus (USB) camera mounted to the device.

### Procedure

Standardized tasks took place within a laboratory. To standardize the use of the app, participants were read a script describing a motor vehicle accident that required immediate and sustained medical attention. They were told that this app was being given to them to monitor their recovery after they left the hospital and they were asked to complete a set of self-report assessments in the coming weeks. Participants used the app a total of 5 times, each time progressing further in their recovery. Trained research assistants observed the participants during their interaction with the app and interactions were recorded with a usability mounted camera [31]. They then completed a brief qualitative interview to assess their thoughts on using the app. Videos were reviewed to identify user interaction errors, defined as errors made by the user due to the interface. These include tapping an icon that is not a responsive icon or being unable to determine how to complete a specific task. The university’s Institutional Review Board approved all procedures.

A clinical psychologist analyzed the qualitative data. A constructivist grounded theory approach was used in which comments and interviews were reviewed multiple times, coded, and primary themes were extracted. Themes that were present for more than three cases were retained. Themes that were present in three or fewer cases were reviewed, merged with other themes, or discarded. Coding and thematic analyses were conducted after each wave to determine the point at which saturation had been obtained and when no new bugs were identified. A hierarchical structure in which themes were evaluated as representing perceived usefulness or perceived ease of use was then evaluated to determine the extent that the qualitative data corresponded to the quantitative data. Several passes of the data determined that this structure represented the data well. Matrix analyses combined the quantitative data from the PUEU and the qualitative data from the interview. Triangulation of the mixed-method yielded a high degree of overlap across the quantitative and qualitative data, which adds validity to the conclusions drawn from the qualitative analysis.

## Results

Participants used the app a total of five times. Participants completed a standard question set that contained 7 items (6 slider-type and 1 toggle-type). Questions assessed symptoms of PTSD (re-experiencing, avoidance, hyperarousal, numbing), pain, and social support, and the presence of trauma-related cues. The time to complete the question sets was mean (SD) 29.37 (7.53) seconds (range 15-57). A review of the video recordings of participant interaction revealed minimal user interaction errors. Participants were able to navigate each use of the app without error. The app stalled for approximately 30 seconds for 2 participants after all responses were logged. No other usability issues were observed. Table 1 shows the results from the qualitative and quantitative data according to the two TAM themes.

**Table 1:** Results of the perceived usefulness and perceived ease of use survey.

		1 (Unlikely)	2	3	4	5	6	7 (Likely)
**Perceived usefulness, %**								
	The app would enable me to communicate with my doctor more quickly.	0.0	4.8	0.0	0.0	28.6	38.1	28.6
	The app would improve my recovery from a traumatic event.	0.0	14.3	9.5	23.8	28.6	19.0	4.8
	The app would improve the quality of medical care I received after a traumatic event.	0.0	4.8	28.6	4.8	28.6	14.3	19.0
	The app would make it easier for me to remember to follow the doctor’s instructions after a traumatic event.	0.0	0.0	4.8	23.8	28.6	23.8	19.0
	The app would make it easier for me to seek additional medical care after a traumatic event.	0.0	4.8	4.8	9.5	33.3	23.8	23.8
	I would find this app useful after a traumatic event.	0.0	4.8	9.5	14.3	28.6	19.0	23.8
**Perceived ease of use, %**								
	Learning to use the app would be easy for me.	0.0	0.0	0.0	9.5	4.8	23.8	61.9
	I would find it easy to get the app to do what I want it to do.	4.8	0.0	0.0	19.0	19.0	14.3	42.9
	My interaction with the app would be clear and understandable.	0.0	0.0	0.0	28.6	14.3	4.8	52.4
	I would find the app to be flexible to interact with.	0.0	4.8	0.0	14.3	23.8	33.3	23.8
	It would be easy for me to become skillful at using the system.	0.0	0.0	0.0	4.8	23.8	19.0	52.4
	I would find the system easy to use.	4.8	0.0	0.0	14.3	9.5	9.5	61.9

### Perceived Usefulness

Ratings of overall perceived usefulness according to the PUEU suggested that participants thought a mobile monitoring system would be useful in improving post-trauma recovery (mean [SD] 5.14 [1.10]). Participants reported the app would facilitate communication with their provider (mean [SD] 5.81 [1.17]). A substantial portion (11/21, 52%) reported that this app would improve communication above and beyond traditional follow-up methods in the qualitative interview. Participants requested two-way communication with their provider through the app (12/21, 57%). That is, they wanted a provider to give feedback, but the type varied. A portion wanted personalized feedback (6/21, 29%), whereas others preferred a notification that the doctor received or viewed their responses (12/21, 57%). Several (3/21, 14%) recommended the app list contact information for a provider. Last, several participants (5/21, 24%) reported that reminders for intervention (eg, take medication, complete physical therapy) would be helpful.

Participants rated the app as moderately likely to improve their recovery from the traumatic event (mean [SD] 4.42 [1.43]) and thought it would be useful after a trauma (mean [SD] 5.19 [1.47]). A majority thought a monitoring system would be helpful (13/21, 62%), with a portion stating it would indicate their provider cared about their recovery (4/21, 19%). However, several participants voiced concerns that this app would replace face-to-face provider contact (2/21, 10%).

### Perceived Ease of Use

Overall ratings suggested the app was easy to use (mean [SD] 5.92 [1.05]; mean calculated out of 7), easy to learn to use (mean [SD] 6.38 [0.97]), and it would be easy to become skillful with the app (mean [SD] 6.19 [0.98]). Qualitative responses were supportive of these data. Nearly all (19/21, 90%) reported the app was easy to use and they enjoyed the simplicity of the design. Several found the design calming and engaging (5/21, 24%). A substantial majority reported assessments took minimal time to complete and would impose minimal burden (17/21, 81%). Indeed, participants reported they would be willing to answer mean (SD) 2.86 (1.85) question sets per day, mean (SD) 4.90 (2.41) days per week.

Participants had several recommendations to enhance the design and features of the app. Half (11/21, 52%) suggested that personalizing the app would be helpful. Specific recommendations included changing colors, setting backgrounds, and personalizing the question content. Personalized content involved using specific details about the individual (eg, name) and questions about their trauma (eg, “how is the pain in your left leg?”). Second, it was recommended that each question have a free text response option to clarify ratings (7/21, 33%).

Participants reported they wanted the app to provide feedback, including a graph of their responses (10/21, 48%). Participants wanted to receive positive feedback that informed them of areas where they were improving and did not wish to be notified if symptoms were worsening (8/21, 38%). Rather, they preferred that worsening outcomes be reported to their provider and the provider contact them.

## Discussion

### Principal Findings

The current study obtained important information about user preferences for a monitoring system for mental health symptoms following a trauma. Participants preferred an app that was easy to use, would not impose a significant burden, and was customizable. The findings are consistent with the TAM [27]. Prior work with websites for health care have also shown that ease of use is correlated with sustained use [32]. The app was focused on a single purpose, obtaining self-report data, which allowed participants to respond to 7 questions in less than 30 seconds on average and showed increased willingness to use the app for a sustained period. The speed with which individuals were able to respond suggests that longer question sets are likely to impose minimal burden. Difficulty providing responses may undermine the utility of mobile monitoring apps [33].

Participants were moderately positive that a mobile monitoring app would help their recovery. This is consistent with evidence suggesting that monitoring is helpful in reducing symptoms of PTSD [34] and that interactions via SMS after a trauma are perceived as helpful [35]. To increase perceived helpfulness, participants should be given a rationale as to the benefit of monitoring. In addition, participants should be told how their data will be used if no other feedback mechanism is available.

A key theme was the importance of providing feedback. Participants were unanimous in their request to interact with their provider through the app rather than as a one-way communication tool. Most users wanted immediate feedback from their provider after completing an assessment. An immediate response, however, would be challenging given the burden this would impose on a provider [12]. Rather, a two-tiered feedback method is recommended. The first would involve an immediate response. This could include a graph of responses, positive praise for completing the assessment, or notification that their provider will review their responses. More patient-specific responses may become feasible as the computational power of mobile devices increases. That is, devices may be able to generate a specific response to a patient based on their answers with a more powerful mobile device. The second type of feedback would involve provider interaction at a later point, such as a phone call or session. Interactions with providers should explicitly highlight that the data obtained from the mobile app triggered this contact. Additional work is needed to determine how to best tailor this feedback and use these data in clinical practice.

A related theme was the personalization of the app to the needs of the patient. Personalized feedback is highly relevant to outcomes and sustained use [36,37]. Advanced analytic methods, such as machine learning, may be especially well suited to provide personalized feedback. These methods can use the large quantities of data generated by these apps to provide specific feedback to an individual [38]. For example, a patient with poor sleep, increased arousal, increased pain, and a prescription for narcotic pain medicine may be at risk for substance abuse. The system could use these data to provide very specific questions or information to the individual about their medication use. Such information would then facilitate care interactions. Relatedly, this ability to tailor question content should specifically address the traumatic event that the participant experienced. Rather than using generic language, it would likely be beneficial to ask targeted questions about specific symptoms, injuries, or events that reference the participant’s experience. Such an approach will assure the participant that this app is tailored to their needs. When implemented successfully, this strategy would improve the efficiency and quality of care for patients in settings with considerable clinical demands, such as the emergency department. As an example, a recent study used mobile telehealth to monitor healing after surgery [39]. The system allowed physicians to monitor healing, provide targeted feedback, and eliminate unnecessary follow-up appointments for those healing as expected while spending more time with those who had complications.

Participants provided two areas of caution. First, participants wanted automated feedback to be positive and preferred that negative outcomes trigger a provider touchpoint. Within the context of TAM, it is possible that negative feedback may diminish the perceived usefulness of an app. Those who are not recovering likely do not want feedback reinforcing their lack of progress but rather want intervention. Alternatively, providing positive feedback about their progress may be perceived as encouraging and supportive. Second, participants cautioned that such monitoring systems should not replace interpersonal care. It is unclear if this concern could be addressed by providing a more personalized experience, such as telehealth or telephone sessions, or additional contact from their provider. Wound care after surgery using telehealth reduced the need for in-person follow-ups, which was preferred by patients [39].

### Limitations

Our conclusions should be considered within the context of several limitations. The sample size for the current study was within the recommended size for usability studies [40] but is still relatively small. The current study was conducted within a laboratory setting with patients who had a trauma history but were not currently dealing with the repercussions of their event. As such, the ecological validity of the current study is limited [26]. Additional usability and feasibility testing is needed with a sample of patients who have recently experienced a traumatic event. Such studies should coincide with validation studies in which responses to the surveys administered via the mobile platform are compared with responses to a gold-standard measure. The majority of the participants in the current study were young, White, female, iPhone-owners, which may limit the generalizability of the findings to other populations. Indeed, recent work has highlighted the ethnic, racial, and economic diversity of patients in an acute setting [15], such that evaluation across a more diverse group of participants is warranted. Finally, the current study focused primarily on the use of a mobile app by patients. The current framework, however, involves a provider dashboard that displays results, allows providers to create and edit question sets, and reviews their patient priorities. Additional usability testing is needed to evaluate this component.

### Conclusion

The results of this study provide several points of feedback to advance modern methods for monitoring mental health recovery after a trauma. The need for personalized feedback, the type of feedback provided, and how patients view such a system has broad implications for other conditions. These recommendations should guide the refinement of current systems and the development of new strategies that leverage novel technology. Although technology changes rapidly, the principles obtained from this study and related projects are applicable to systems that address mental health. Such work is essential to the development of systems that will be used by patients to improve outcomes.

## Abbreviations

PTSD: posttraumatic stress disorder
PUEU: Perceived Useful and Ease of Use Survey
TAM: Technology Acceptance Model
